# Supplementary material for: Pattern decorrelation in the mouse medial prefrontal cortex enables social preference and requires MeCP2
Source: Nat Commun. 2022 Jul 6;13:3899. doi: 10.1038/s41467-022-31578-9 (PMC9259602; doi:10.1038/s41467-022-31578-9)
Supplement: Supplementary file 1 — Supplementary Information [file 41467_2022_31578_MOESM1_ESM.pdf]

Supplementary Information

Social preference requires pattern decorrelation in the medial prefrontal cortex

Pan Xu, Yuanlei Yue, Juntao Su, Xiaoqian Sun, Hongfei Du, Zhichao Liu, Rahul Simha, Jianhui Zhou, Chen Zeng, Hui Lu

Supplementary material includes:

Figures S1-S10

Movies S1-S4

Table 1

Supplementary Figures and Legends:

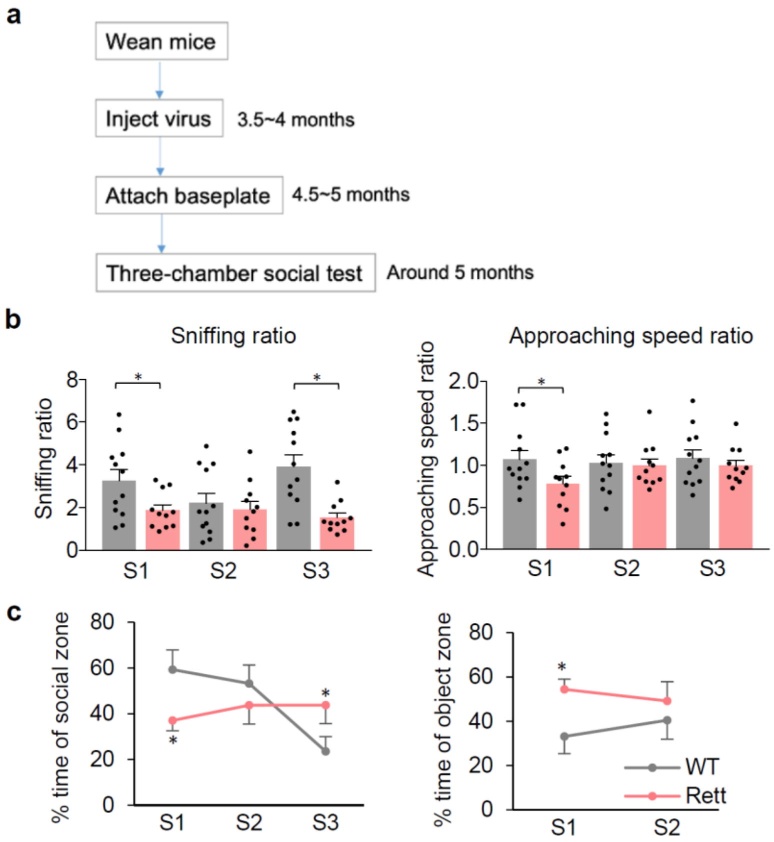

Supplementary Figure 1. Five-month old female WT, but not *Mecp2*$^{+/-}$ mice ("Rett mice"), show a preference for social interactions over investigating inanimate objects.

a Workflow of the surgical and testing experiments.

b The sniffing time ratio (left) and the approaching speed ratio of M1 to O in S1, S2 (M2 to M1 in S3) (right) in the WT and Rett mice.

c Percentage of time (out of 10 minute session) WT and Rett mice spent in interaction with M1 (left) and O (right) across sessions.

Data are represented as mean ± SEM. $*P < 0.05$, Rett (n=11) mice v.s. WT (n=12), two-way RM ANOVA with Bonferroni-corrected post hoc comparisons.

Source data are provided as a Source Data file.

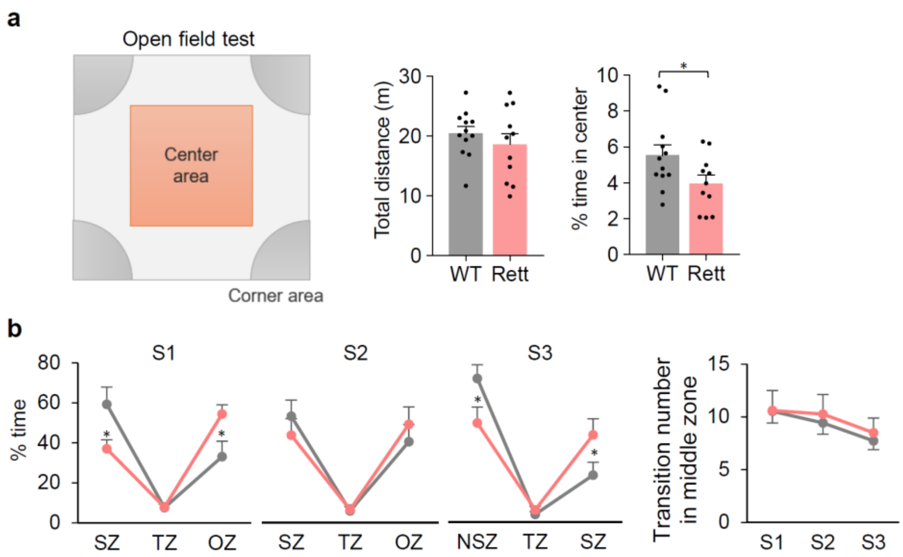

Supplementary Figure 2. Motor activity and transitions across the middle zone did not influence the social interaction test.

a *Left*: Diagram of open field test (OFT) chamber. *Right*: The averaged total distance of the movement in the chamber for 10 minutes and the percentage of time spent in the center area by WT (n=12) and Rett (n=11) mice at 5 months of age. The motor activity of Rett mice is equivalent to WT at this stage, though Rett mice are much more anxious.

b Time percentage mice spent in different zones (left) and transition number through middle area (right). Rett mice (n=11) spent much less time in the social zone (SZ) or new social zone (NSZ) than WT mice (n=12), whereas both genotypes spent similar time in the transition zone (TZ) and the number of times they traversed the chamber throughout the three sessions. SZ, NSZ, and object zone (OZ) indicate the 10-cm ends of the central chamber close to M1, M2, and O. *$P < 0.05$, two-way RM ANOVA with Bonferroni-corrected post hoc comparisons.

Source data are provided as a Source Data file.

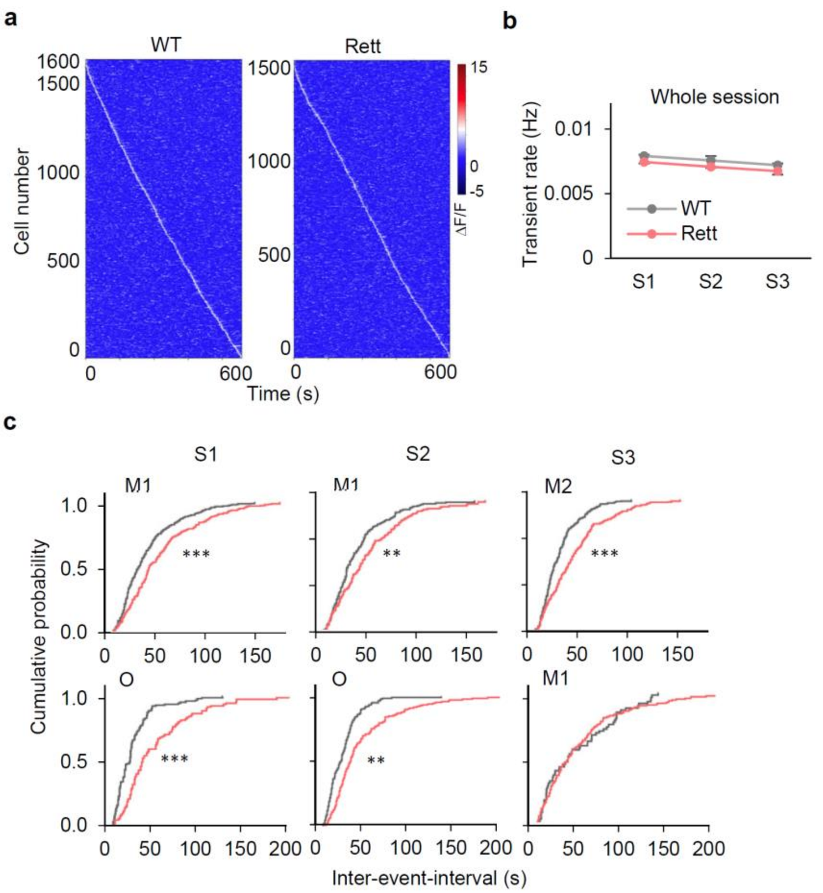

Supplementary Figure 3. Recording mPFC activity in WT and Rett mice.

a Raster plots of calcium activity of individual mPFC neurons over the whole testing session from one representative WT and one representative Rett mouse.

b Averaged mPFC transient rate of a 10-min testing session from WT (n=9) and Rett (n=8) mice. No significant difference was found between genotypes, two-way RM ANOVA with Bonferroni-corrected post hoc comparisons.

c The cumulative probability of inter-event intervals in mPFC neurons when WT and Rett mice were interacting with stimuli in each session. ***P* <0.01, ****P* <0.001, two-sided Kolmogorov–Smirnov (KS) test.

Source data are provided as a Source Data file.

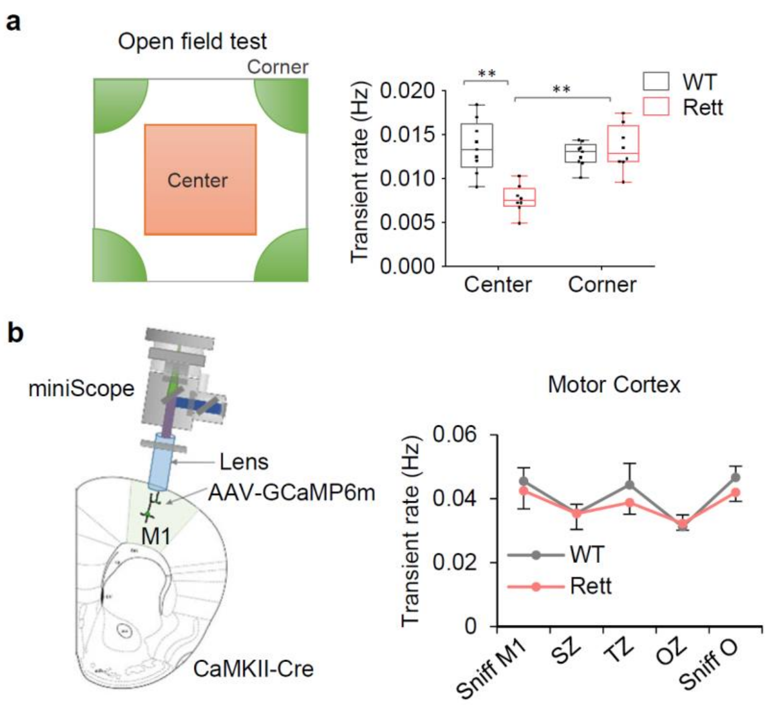

Supplementary Figure 4. Neural activity in the mPFC during the Open Field Test and in the motor cortex during the three-chamber test.

a *Left*: Diagram of open field test (OFT) chamber. *Right*: Averaged transient rate of recorded mPFC population when WT (n=9) and Rett (n=8) mice were in the center and corner areas of the OFT. Error bars indicate SEM. ***P* <0.01, two-way RM ANOVA with Bonferroni-corrected post hoc comparisons.

b *Left*: Diagram showing virus injection and lens implantation in the motor cortex (M1) for imaging pyramidal neurons in CaMKII-Cre mice with a miniscope. *Right*: The transient rate of motor cortex in WT and Rett mice across different locations in the chamber. "Sniff" means the mouse was at the end of the chamber and interacting with a stimulus; social zone (SZ), object zone (OZ) indicate the 10-cm regions of the central chamber that are nearest to the social or object stimulus; transition zone (TZ) means the middle area in the central chamber. WT: n=9 mice; Rett: n=8 mice. Data are represented as mean ± SEM. There was no significant difference between the two genotypes; two-way RM ANOVA with Bonferroni-corrected post hoc comparisons.

Source data are provided as a Source Data file.

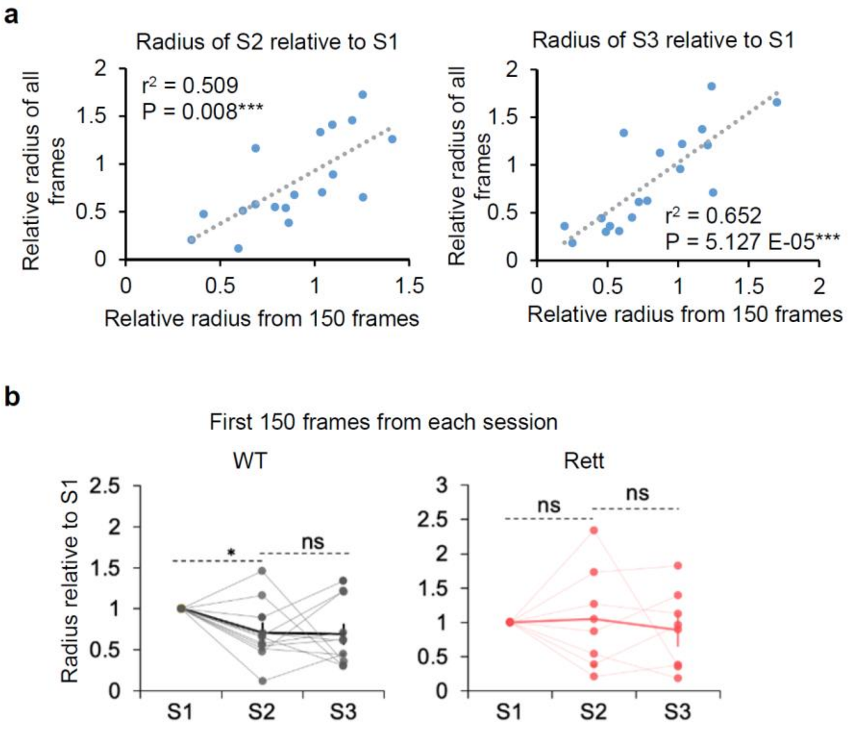

Supplementary Figure 5. VAE analysis was consistent regardless of frame number.

a Pearson's correlation between relative distribution radii (normalized to S1) based on the first 150 "social interaction" frames and those based on all "social interaction" frames. Each dot indicates the relative radius of one mouse based on two different frames. All mice were included (n=18). Pearson's correlations were calculated across genotypes. ***$P < 0.001$, regression.

b Changes of relative distribution radius (compared to S1) across three sessions averaged from the first 150 "social interaction" frames of each individual WT (n=11) and Rett (n=7) mouse. Each dot indicates the normalized averaged values. Lines connect data points from the same mouse. The thicker solid lines connect the averaged value from all the mice of each group. Error bars indicate SEM. *$P < 0.05$, ns, no significance, two-way RM ANOVA with Bonferroni-corrected post hoc comparisons.

Source data are provided as a Source Data file.

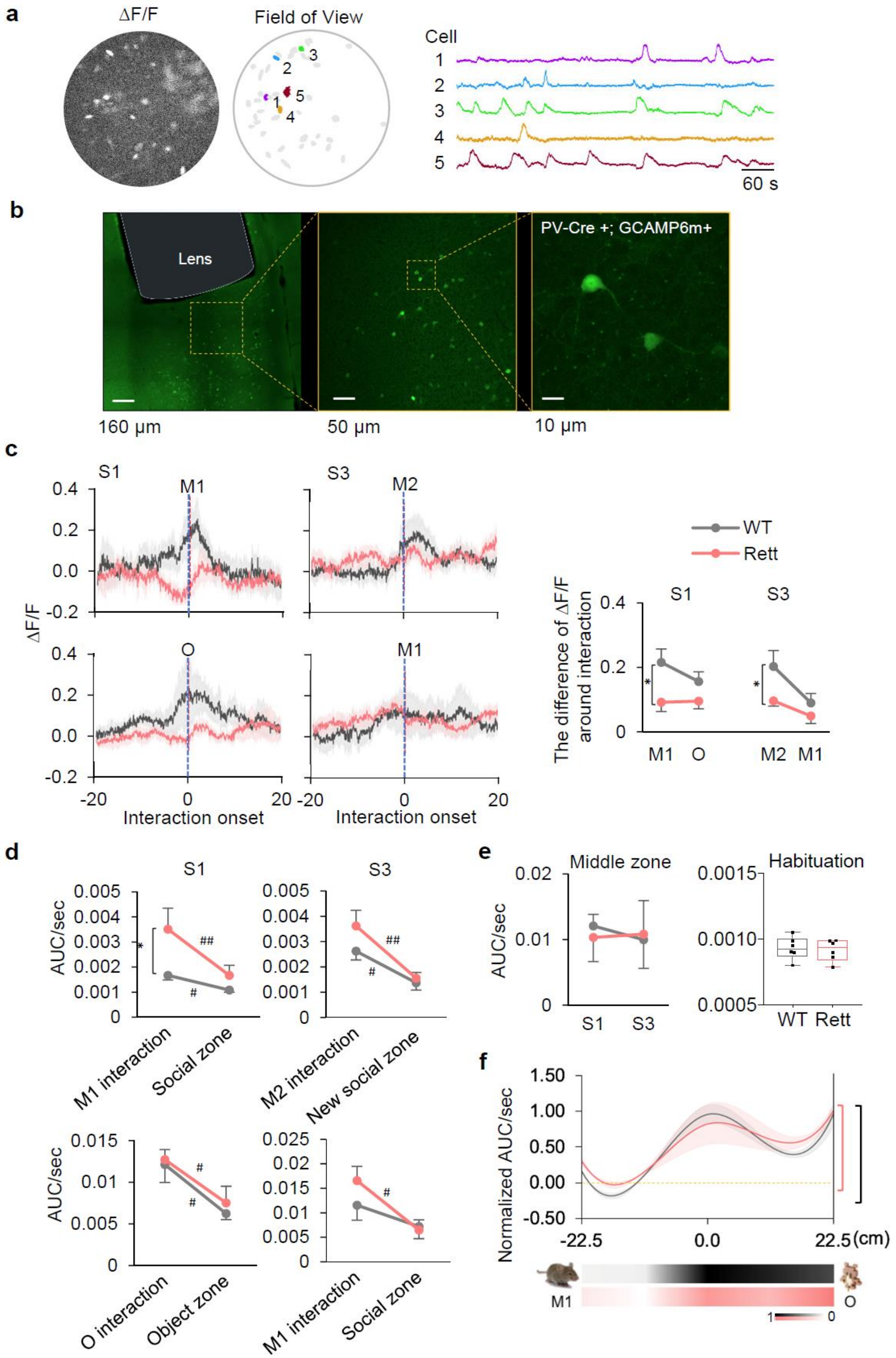

a
ΔF/F
Field of View
Cell
1
2
3
4
5
60 s
b
Lens
PV-Cre +; GCAMP6m+
160 μm
50 μm
10 μm
c
S1
M1
S3
M2
O
M1
ΔF/F
0.4
0.2
0.0
-0.2
-20
0
20
Interaction onset
WT
Rett
The difference of ΔF/F around interaction
M1
O
M2
M1
d
S1
S3
AUC/sec
M1 interaction
Social zone
M2 interaction
New social zone
O interaction
Object zone
M1 interaction
Social zone
e
Middle zone
Habituation
AUC/sec
S1
S3
WT
Rett
f
Normalized AUC/sec
-22.5
0.0
22.5
(cm)
M1
O

Supplementary Figure 6. PV inhibitory neurons in Rett mice are hyperactive in responding to stimuli

a *Left*: the ΔF/F image under a GRIN lens in one mouse and the field of view with identified PV interneurons, of which five were numbered and colored. *Right*: fluorescence traces of the five example neurons marked in the left panel.

b Representative image (selected from experiments performed in 12 mice) of the viral expression in PV interneurons of prelimbic region with GRIN lens implantation.

c *Left*: averaged calcium traces of PV interneurons around the onset of interactions in S1 and S3 (20 s before to 20 s after). Solid lines and shaded regions represent the averaged value and SEM, respectively. *Right*: amplitude increases of PV interneurons around the onset of interactions (20 s after minus 20 s before). ΔF/F=change in fluorescence over baseline fluorescence intensity. Values are represented as mean ± SEM. *$P < 0.05$, Rett (n=6) vs. WT (n=6), two-way ANOVA with Bonferroni-corrected post hoc comparisons.

d, e The AUC (area under curve) of calcium traces per second of the WT (n=6) and Rett (n=6) mice when they were interacting or near the end chamber in S1 and S3 (**d**), as well as when they were traversing the middle zone or habituating before the test (**e**). Values are represented as mean ± SEM. *$P < 0.05$, Rett vs. WT; #$P < 0.05$, ##$P < 0.01$, interaction vs. zone, two-way ANOVA with Bonferroni-corrected post hoc comparisons. Box boundaries are the 25th and 75th percentiles, the horizontal line across the box is the median, the cross "+" indicates the mean value, and the whiskers indicate the minimum and maximum values.

f The response field of PV neurons based on the the AUC per second of PV activity traces. The average of normalized AUC/sec of WT and Rett mice are presented as smoothed curves, with standard error (SEM) conveyed by shaded regions. Below is a heatmap of the spatial field of the averaged AUC/sec of PV neurons in WT mice. The darker color indicated higher value.

Source data are provided as a Source Data file.

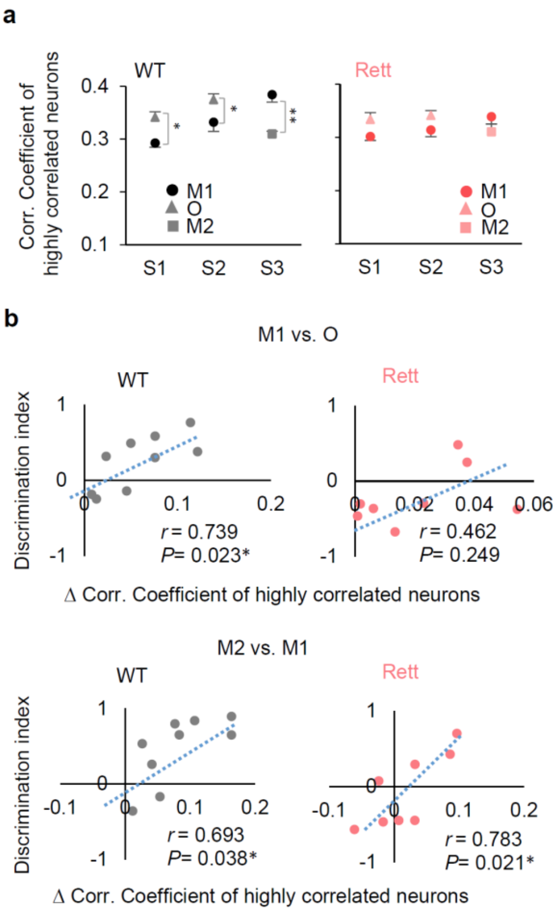

Supplementary Figure 7. Relationships of the most highly correlated neuronal pairs in WT and Rett mPFC.

a The averaged Pearson's correlation coefficient of highly correlated neuronal pairs in the WT (n=9) and Rett (n = 8) mice during different interactions. High correlation: |correlation coefficient| > 0.2. Error bars indicate SEM. **P* < 0.05, ***P* < 0.01, two-way RM ANOVA with Bonferroni-corrected post hoc comparisons.

b Correlation between the correlation coefficient difference of highly correlated neurons and the discrimination index of M1 vs. O (top) and M2 vs. M1 (bottom) in WT (n=9) and Rett (n=8) mice. Each dot represents an individual mouse; Pearson's correlation coefficients were calculated across genotypes. ***P* < 0.01, ****P* < 0.001, regression.

Source data are provided as a Source Data file.

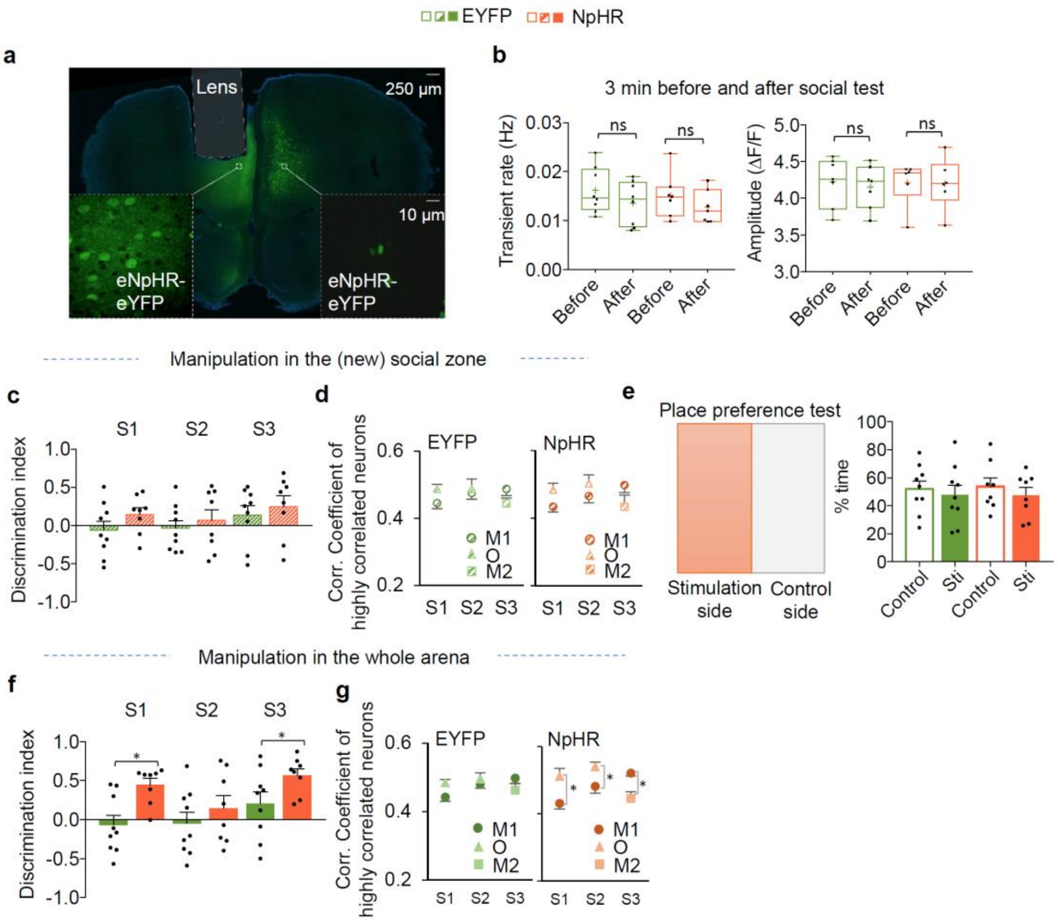

Supplementary Figure 8. Whole-arena optogenetic stimulation of mPFC restores social preference in Rett mice.

a Histology of PL with GRIN lens implantation and viral expression in pyramidal neurons (left square) and PV neurons (right square). Representative image are selected from experiments performed in 15 mice.

b The mPFC transient rate and amplitude for 3-min periods before and after social interaction tests in NpHR (n=8) and EYFP (n=7) WT mice. No significant difference between NpHR and EYFP group was found; two-way RM ANOVA with Bonferroni-corrected post hoc comparisons. Box boundaries are the 25th and 75th percentiles, the horizontal line across the box is the median, the cross "+" indicates the mean value, and the whiskers indicate the minimum and maximum values.

c, d Manipulation of the mPFC solely within the (new) social-zone did not significantly influence the discrimination indices **(c)** or the correlation coefficients of highly correlated neuronal pairs **(d)** of NpHR (n=9) mice, compared with EYFP (n=8) mice. High correlation: |correlation coefficient| > 0.2. Error bars indicate SEM. *$P < 0.05$, two-way RM ANOVA with Bonferroni-corrected post hoc comparisons.

e *Left*: Place preference testing, with stimulation given only when the mouse moves into the stimulation side (orange). *Right*: the percentage of time NpHR (n = 9) and EYFP (n = 8) mice spent in each side. Error bars indicate SEM. There was no significant difference between groups or conditions (two-way ANOVA with Bonferroni-corrected post hoc comparisons).

f, g Manipulation of the mPFC throughout the 10-min session (not just during social interaction) significantly increased the discrimination indices **(e)** and the difference in correlation coefficient of highly correlated neuron pairs between two lateral stimuli **(f)** of NpHR (n = 9) mice, compared with EYFP (n = 8) mice. High correlation: |correlation coefficient| > 0.2. Error bars indicate SEM. *P < 0.05, two-way RM ANOVA with Bonferroni-corrected post hoc comparisons.

Source data are provided as a Source Data file.

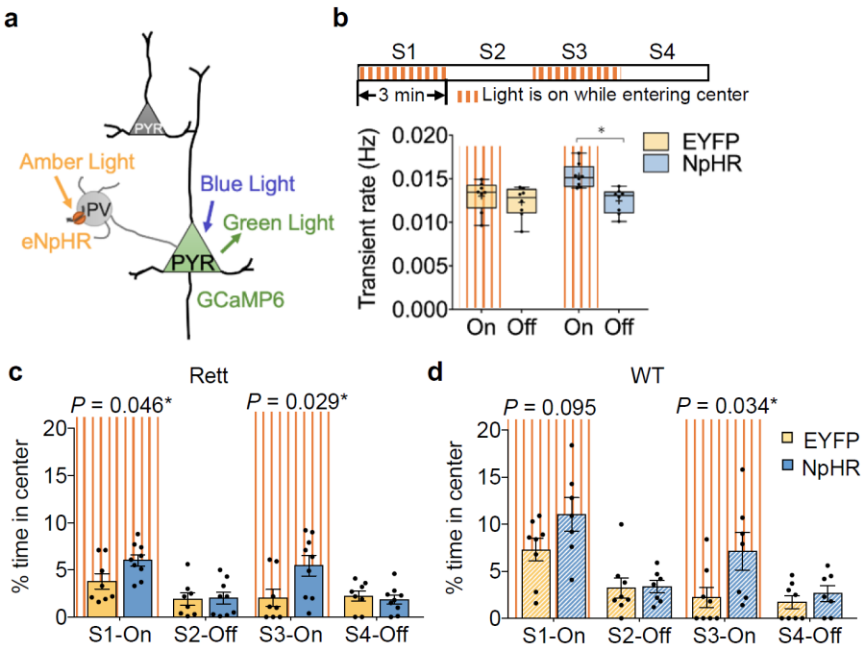

Supplementary Figure 9. Elevating the activity of mPFC excitatory neurons in the anxiety-provoking center zone is sufficient to relieve anxiety.

a Schematic of light path for the head-mounted microscope enabling optogenetic parvalbumin (PV) suppression and simultaneous imaging of pyramidal neurons (PNs) in the PL region of the *PV-Cre* mice. Inhibitory halorhodopsin NpHR (control protein, EYFP) and GCaMP6m were expressed by PV interneurons and excitatory PNs, respectively.

b Averaged transient rate of NpHR (n = 9) and EYFP (n = 8) mice in four three-min trials with or without PL optogenetic manipulation (S1-S4). Values were plotted as mean ± SEM. *P <0.05, amber light On versus Off, two-way ANOVA with Bonferroni-corrected post hoc comparisons. Box boundaries are the 25th and 75th percentiles, the horizontal line across the box is the median, the cross "+" indicates the mean value, and the whiskers indicate the minimum and maximum values.

c, d Optogenetic mPFC manipulation with light reduced the anxiety-like performance in NpHR-expressing Rett (**c**) and WT (**d**) mice, who spent more time in the center area than EYFP mice. Error bars indicate SEM. *P <0.05, NpHR (n = 9) vs. EYFP (n = 8), two-way ANOVA with Bonferroni-corrected post hoc comparisons.

Source data are provided as a Source Data file.

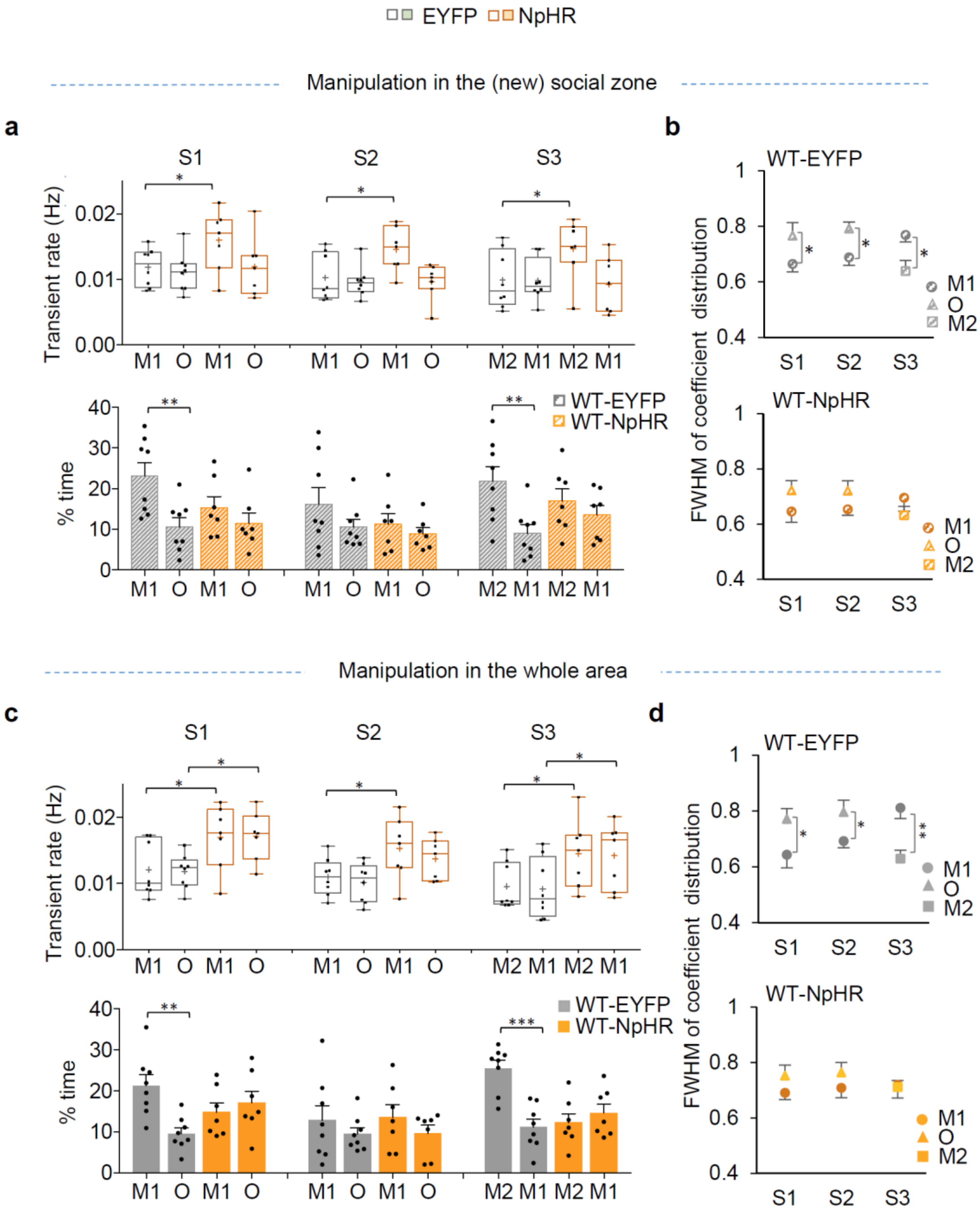

Supplementary Figure 10. Elevating the activity of mPFC pyramidal neurons by suppressing PV interneurons impairs the sociability and social novelty preference of WT mice.

a Suppressing PV interneurons only in the social zone elevated the transient rate of the mPFC and reduced the social preference and social novelty preference of WT mice (top). Box boundaries are the 25th and 75th percentiles, the horizontal line across the box is the median, the cross "+" indicates the mean value, and the whiskers indicate the minimum and maximum values. *$P < 0.05$, **$P < 0.01$, EYFP (n=8) vs. NpHR (n=7), two-way ANOVA with post hoc Bonferroni correction.

b The averaged FWHM of correlation distribution of WT mice expressing EYFP (n=8) and NpHR (n=7) during interactions with different stimuli. Data are represented as mean ± SEM. *$P < 0.05$, two-way RM ANOVA with Bonferroni-corrected post hoc comparisons.

c The whole-arena excitation of mPFC impaired the social preference in S1 and social novelty preference in S3 of NpHR mice (n=7), compared with EYFP mice (n=8). Box boundaries are the 25th and 75th percentiles, the horizontal line across the box is the median, the cross "+" indicates the mean value, and the whiskers indicate the minimum and maximum values. *$P < 0.05$, **$P < 0.01$, ***$P < 0.001$, two-way ANOVA with post hoc Bonferroni correction.

d Whole-arena manipulation of mPFC impaired pattern decorrelation in WT mice expressing EYFP (n=8) and NpHR (n=7), as indicated by the narrower spread between FWHM values in all three sessions. *$P < 0.05$, **$P < 0.01$, two-way ANOVA with Bonferroni-corrected post hoc comparisons.

Source data are provided as a Source Data file.

Supplementary Movies

Movie S1. Female WT mouse prefers interacting with other mouse (M1) over object (O) during session 1.

Movie S2: Female *Mecp2+/-* mice lack the usual murine preference for interacting with other mouse (M1) over object (O) during session 1.

Movie S3. Female WT mouse prefers interacting with the new mouse (M2) over the old mouse (M1) during session 3.

Movie S4: Female Mecp2+/- mice lack the usual preference for interacting with a new mouse (M2) over the old mouse (M1) during session 3.

Supplemental table 1: Detailed statistical results for each experiment, organized by figure panel.

Figure. Panel	Test type	Sample size (mice)	Subgroup	Test stat.	DF	Comparison	P-value	Asterisk
Fig. 1c left	Two-way ANOVA	WT: 12; Rett: 11	S1	F = 6.404	dfn=1, dfd=42	WT, M1 vs. O	9.4 E-5	***
						Rett, M1 vs. O	0.609	ns
			S2	F = 1.816	dfn=1, dfd=42	WT, M1 vs. O	0.038	*
						Rett, M1 vs. O	0.366	ns
			S3	F = 3.463	dfn=1, dfd=42	WT, M1 vs. M2	0.004	**
						Rett, M1 vs. M2	0.695	ns
Fig. 1c right	Two way RM-ANOVA	WT: 12; Rett: 11	S1, S2, S3	F = 11.377	dfn=1, dfd=10	WT vs. Rett, S1	0.028	*
						WT vs. Rett, S2	0.510	ns
						WT vs. Rett, S3	0.013	*
Fig. 2d	Two-way ANOVA	WT: 9; Rett: 8	S1	F = 14.002	dfn=1, dfd=30	WT vs. Rett, M1	0.022	*
						WT vs. Rett, O	0.016	*
			S2	F = 7.119	dfn=1, dfd=30	WT vs. Rett, M1	0.020	*
						WT vs. Rett, O	0.183	ns
			S3	F = 4.196	dfn=1, dfd=30	WT vs. Rett, M2	0.028	*
						WT vs. Rett, M1	0.541	ns
Fig. 2f	Two-way ANOVA	WT: 9; Rett: 8	WT/Rett	F = 0.715	dfn=1, dfd=30	WT vs. Rett, left	0.980	ns
						WT vs. Rett, right	0.973	ns
Fig. 3a	Two way RM-ANOVA	WT: 9; Rett: 8	S1, S2, S3	F = 5.153	dfn=1, dfd=7	WT vs. Rett, S1	0.048	*
						WT vs. Rett, S2	0.018	*
						WT vs. Rett, S3	0.550	ns
Fig. 3b	Two way RM-ANOVA	WT: 9; Rett: 8	S1, S2, S3	F = 3.392	dfn=1, dfd=7	WT, S1 vs. S2	0.015	*
						WT, S2 vs. S3	0.014	*
						Rett, S1 vs. S2	0.517	ns
						Rett, S2 vs. S3	0.319	ns

Fig. 3d	Two way RM-ANOVA	WT: 11; Rett: 7	S1, S2, S3	F = 0.634	dfn=1, dfd=6	WT, S1 vs. S3	0.047	*
						WT, S1 vs. S2	0.032	*
						WT, S2 vs. S3	0.307	ns
						Rett, S1 vs. S3	0.663	ns
						Rett, S1 vs. S2	0.823	ns
						Rett, S2 vs. S3	0.974	ns
Fig. 4c	Two way-ANOVA	WT: 9	Stimuli	F = 14.101	dfn=2, dfd=48	M1vs. O	0.024	*
						M1vs. O	0.030	*
						M2 vs. M1	0.002	**
		Rett: 8	Stimuli	F = 2.642	dfn=2, dfd=42	M1vs. O	0.492	ns
						M1vs. O	0.286	ns
						M2 vs. M1	0.295	ns
Fig. 5d, %time	Two-way ANOVA	EYFP: 8; NpHR: 9	S1	F = 0.385	dfn=1, dfd=30	EYFP, M1 vs. O	0.791	ns
						NpHR, M1 vs. O	0.321	ns
			S2	F = 0.109	dfn=1, dfd=30	EYFP, M1 vs. O	0.728	ns
						NpHR, M1 vs. O	0.225	ns
			S3	F = 0.768	dfn=1, dfd=30	EYFP, M1 vs. M2	0.388	ns
						NpHR, M1 vs. M2	0.695	ns
Fig. 5d, transient rate	Two-way ANOVA	EYFP: 8; NpHR: 9	S1	F = 5.008	dfn=1, dfd=30	EYFP vs. NpHR, M1	0.011	*
						EYFP vs. NpHR, O	0.648	ns
			S2	F = 2.453	dfn=1, dfd=30	EYFP vs. NpHR, M1	0.037	*
						EYFP vs. NpHR, O	0.636	ns
			S3	F = 2.181	dfn=1, dfd=30	EYFP vs. NpHR, M2	0.018	*
						EYFP vs. NpHR, M1	0.674	ns
Fig. 5e	Two way-ANOVA	EYFP: 8	Stimuli	F = 0.023	dfn=2, dfd=42	M1vs. O	0.423	ns
						M1vs. O	0.687	ns
						M2 vs. M1	0.311	ns
		NpHR: 9	Stimuli	F = 0.107	dfn=2, dfd=48	M1vs. O	0.193	ns
						M1vs. O	0.213	ns
						M2 vs. M1	0.438	ns
Fig. 5g, %time	Two-way ANOVA	EYFP: 8; NpHR: 9	S1	F = 4.662	dfn=1, dfd=30	EYFP vs. NpHR, O	0.833	ns
						EYFP vs. NpHR, M1	0.012	*

			S2	F = 1.035	dfn=1, dfd=30	EYFP vs. NpHR, O	0.702	ns
						EYFP vs. NpHR, M1	0.048	*
			S3	F = 4.273	dfn=1, dfd=30	EYFP vs. NpHR, M1	0.184	ns
						EYFP vs. NpHR, M2	0.018	*
Fig. 5g, transient rate	Two-way ANOVA	EYFP: 8; NpHR: 9	S1	F = 7.941	dfn=1, dfd=30	EYFP vs. NpHR, M1	0.037	*
						EYFP vs. NpHR, O	0.014	*
			S2	F = 16.81	dfn=1, dfd=30	EYFP vs. NpHR, M1	0.003	**
						EYFP vs. NpHR, O	0.016	*
			S3	F = 8.772	dfn=1, dfd=30	EYFP vs. NpHR, M2	0.020	*
						EYFP vs. NpHR, M1	0.095	ns
Fig. 5h	Two way-ANOVA	EYFP: 8	Stimuli	F = 0.014	dfn=2, dfd=42	M1vs. O	0.523	ns
						M1vs. O	0.467	ns
						M2 vs. M1	0.121	ns
		NpHR: 9	Stimuli	F = 33.986	dfn=2, dfd=48	M1vs. O	0.001	**
						M1vs. O	0.013	*
						M2 vs. M1	0.004	**
Fig. 6b, transient rate	Two-way ANOVA	EYFP: 6; NpHR: 6	EYFP/NpHR	F = 2.485	dfn=1, dfd=20	EYFP, ON vs. OFF	0.697	ns
						NpHR, ON vs. OFF	0.016	*
Fig. 6b, amplitude	Two-way ANOVA	EYFP: 6; NpHR: 6	EYFP/NpHR	F = 0.1572	dfn=1, dfd=20	EYFP, ON vs. OFF	0.697	ns
						NpHR, ON vs. OFF	0.353	ns
Fig. 6c, %time	Two-way ANOVA	EYFP: 6; NpHR: 6	S1	F = 17.030	dfn=1, dfd=20	EYFP, M1 vs. O	0.000	***
						NpHR, M1 vs. O	0.316	ns
			S2	F = 3.266	dfn=1, dfd=20	EYFP, M1 vs. O	0.145	ns
						NpHR, M1 vs. O	0.310	ns
			S3	F = 2.712	dfn=1, dfd=20	EYFP, M1 vs. M2	0.048	*
						NpHR, M1 vs. M2	0.826	ns
Fig. 6c, transient rate	Two-way ANOVA	EYFP: 6; NpHR: 6	S1	F = 2.345	dfn=1, dfd=20	EYFP vs. NpHR, M1	0.041	*
						EYFP vs. NpHR, O	0.974	ns

			S2	F = 2.093	dfn=1, dfd=20	EYFP vs. NpHR, M1	0.049	*
						EYFP vs. NpHR, O	0.774	ns
			S3	F = 2.379	dfn=1, dfd=20	EYFP vs. NpHR, M2	0.045	*
						EYFP vs. NpHR, M1	0.767	ns
Fig. 6d	Two way-ANOVA	EYFP: 6	Stimuli	F = 0.848	dfn=2, dfd=30	M1vs. O	0.026	*
						M1vs. O	0.029	*
						M2 vs. M1	0.047	*
		NpHR: 6	Stimuli	F = 0.2779	dfn=2, dfd=30	M1vs. O	0.829	ns
						M1vs. O	0.607	ns
						M2 vs. M1	0.776	ns
Fig. 6e, %time	Two-way ANOVA	EYFP: 6; NpHR: 6	S1	F = 12.643	dfn=1, dfd=20	EYFP, M1 vs. O	0.000	***
						NpHR, M1 vs. O	0.653	ns
			S2	F = 0.017	dfn=1, dfd=20	EYFP, M1 vs. O	0.229	ns
						NpHR, M1 vs. O	0.170	ns
			S3	F = 8.281	dfn=1, dfd=20	EYFP, M1 vs. M2	0.003	**
						NpHR, M1 vs. M2	0.498	ns
Fig. 6e, transient rate	Two-way ANOVA	EYFP: 6; NpHR: 6	S1	F = 8.556	dfn=1, dfd=20	EYFP vs. NpHR, M1	0.031	*
						EYFP vs. NpHR, O	0.045	*
			S2	F = 10.546	dfn=1, dfd=20	EYFP vs. NpHR, M1	0.031	*
						EYFP vs. NpHR, O	0.035	*
			S3	F = 6.802	dfn=1, dfd=20	EYFP vs. NpHR, M2	0.043	*
						EYFP vs. NpHR, M1	0.118	ns
Fig. 6f	Two way-ANOVA	EYFP: 6	Stimuli	F = 0.584	dfn=2, dfd=30	M1vs. O	0.021	*
						M1vs. O	0.015	*
						M2 vs. M1	0.025	*
		NpHR: 6	Stimuli	F = 0.412	dfn=2, dfd=30	M1vs. O	0.765	ns
						M1vs. O	0.991	ns
						M2 vs. M1	0.565	ns

Supplementary Fig. 1b left	Two way RM-ANOVA	WT: 12; Rett: 11	S1, S2, S3	F = 19.636	dfn=1, dfd=10	WT vs. Rett, S1	0.038	*
						WT vs. Rett, S2	0.510	ns
						WT vs. Rett, S3	0.031	*

Supplementary Fig. 1b right	Two way RM-ANOVA	WT: 12; Rett: 11	S1, S2, S3	F = 1.361	dfn=1, dfd=10	WT vs. Rett, S1	0.022	*
						WT vs. Rett, S2	0.510	ns
						WT vs. Rett, S3	0.423	ns
Supplementary Fig. 1c, left	Two way RM-ANOVA	WT: 12; Rett: 11	S1, S2, S3	F = 0.835	dfn=1, dfd=10	WT vs. Rett, S1	0.038	*
						WT vs. Rett, S2	0.303	ns
						WT vs. Rett, S3	0.013	*
Supplementary Fig. 1c, right	Two way RM-ANOVA	WT: 12; Rett: 11	S1, S2	F = 4.567	dfn=1, dfd=10	WT vs. Rett, S1	0.019	*
						WT vs. Rett, S2	0.385	ns
Supplementary Fig. 2a left	T test	WT: 11; Rett: 11	no	t=0.692	df =20	WT vs. Rett	0.812	ns
Supplementary Fig. 2a right	T test	WT: 11; Rett: 11	no	t =6.972	df =20	WT vs. Rett	0.042	*
Supplementary Fig. 2b left	Two-way ANOVA	WT: 12; Rett: 11	S1	F = 14.441	df1=5, df2=64	SZ, WT vs. Rett	0.012	*
						TZ, WT vs. Rett	0.870	ns
						OZ, WT vs. Rett	0.020	*
			S2	F = 11.995	df1=5, df2=64	SZ, WT vs. Rett	0.276	ns
						TZ, WT vs. Rett	0.863	ns
						OZ, WT vs. Rett	0.244	ns
			S3	F = 19.120	df1=5, df2=64	SZ, WT vs. Rett	0.014	*
						TZ, WT vs. Rett	0.714	ns
						OZ, WT vs. Rett	0.020	*
Supplementary Fig. 2b right	Two way RM-ANOVA	WT: 12; Rett: 11	S1, S2, S3	F = 1.288	df1=5, df2=64	WT vs. Rett, S1	0.538	ns
						WT vs. Rett, S2	0.277	ns
						WT vs. Rett, S3	0.368	ns
Supplementary Fig. 3b	Two way RM-ANOVA	WT: 12; Rett: 11	S1, S2, S3	F = 0.799	df1=5, df2=45	WT vs. Rett, S1	0.753	ns
						WT vs. Rett, S2	0.855	ns
						WT vs. Rett, S3	0.204	ns
Supplementary Fig. 4a	Two-way ANOVA	WT: 9; Rett: 8	WT/Rett	F = 10.26	dfn=1, dfd=30	WT vs. Rett, center	0.004	**
						WT vs. Rett, corner	0.914	ns

			center/corner	F = 11.38	dfn=1, dfd=30	center vs. corner, WT	0.852	ns
						center vs. corner, Rett	0.002	**
Supplementary Fig. 4b	Two way-ANOVA	WT: 4; Rett: 4	5 zones	F = 1.389	dfn=1, dfd=30	M1: WT vs. Rett	0.659	ns
						SZ: WT vs. Rett	0.987	ns
						TZ: WT vs. Rett	0.413	ns
						OZ: WT vs. Rett	0.882	ns
						O: WT vs. Rett	0.490	ns
Supplementary Fig. 5b	Two way RM-ANOVA	WT: 11; Rett: 7	S1, S2, S3	F = 0.597	dfn=1, dfd=6	WT, S1 vs. S2	0.031	*
						WT, S2 vs. S3	0.392	ns
						Rett, S1 vs. S2	0.682	ns
						Rett, S2 vs. S3	0.587	ns
Supplementary Fig. 6c	Two-way ANOVA	WT: 6; Rett: 6	S1	F = 11.39	dfn=1, dfd=20	M1, WT vs. Rett	0.042	*
						O, WT vs. Rett	0.316	ns
			S3	F = 8.171	dfn=1, dfd=20	M2, WT vs. Rett	0.048	*
						M1, WT vs. Rett	0.254	ns
Supplementary Fig. 6d	Two-way ANOVA	WT: 6; Rett: 6	S1, M1 side	F = 6.643	dfn=1, dfd=20	M1, WT vs. Rett	0.048	*
						social zone, WT vs. Rett	0.897	ns
				F = 6.645	dfn=1, dfd=20	WT, M1 vs. zone	0.008	#
						Rett, M1 vs. zone	0.049	##
			S1, O side	F = 13.85	dfn=1, dfd=20	O, WT vs. Rett	0.998	ns
						object zone, WT vs. Rett	0.718	ns
				F = 0.398	dfn=1, dfd=20	WT, O vs. zone	0.047	#
						Rett, O vs. zone	0.010	#
			S3, M2 side	F = 17.15	dfn=1, dfd=20	M2, WT vs. Rett	0.320	ns
						new social zone, WT vs. Rett	0.991	ns
				F = 2.172		WT, M2 vs. zone	0.035	#

					dfn=1, dfd=20	Rett, M2 vs. zone	0.0081	##
			S3, M1 side	F = 10.15	dfn=1, dfd=20	M1, WT vs. Rett	0.361	ns
						social zone, WT vs. Rett	0.702	ns
				F = 0.398	dfn=1, dfd=20	WT, M1 vs. zone	0.336	ns
						Rett, M1 vs. zone	0.042	#
Supplementary Fig. 6e left	Two-way ANOVA	WT: 6; Rett: 6	session	F = 0.065	dfn=1, dfd=20	M1, WT vs. Rett	0.978	ns
						O, WT vs. Rett	0.997	ns
Supplementary Fig. 6e right	t test	WT: 6; Rett: 6	no	0.336	df = 10	WT vs. Rett	0.902	ns
Supplementary Fig. 7a	Two way-ANOVA	WT: 9	Stimuli	F = 3.159	dfn=2, dfd=48	M1vs. O	0.019	*
						M1vs. O	0.023	*
						M2 vs. M1	0.005	**
		Rett: 8	Stimuli	F = 2.642	dfn=2, dfd=42	M1vs. O	0.392	ns
						M1vs. O	0.476	ns
						M2 vs. M1	0.253	ns
Supplementary Fig. 8b, transient rate	Two-way ANOVA	NpHR: 9; EYFP: 8	EYFP/NpHR	F = 0.692	dfn=1, dfd=30	EYFP, ON vs. OFF	0.915	ns
						NpHR, ON vs. OFF	0.002	**
Supplementary Fig. 8b, amplitude	Two-way ANOVA	NpHR: 9; EYFP: 8	EYFP/NpHR	F = 1.647	dfn=1, dfd=30	EYFP, ON vs. OFF	0.496	ns
						NpHR, ON vs. OFF	0.936	ns
Supplementary Fig. 8c, transient rate	Two-way ANOVA	NpHR: 9; EYFP: 8	before/after	F = 0.367	dfn=1, dfd=30	EYFP, before vs. after	0.511	ns
						NpHR, before vs. after	0.852	ns
Supplementary Fig. 8c, amplitude	Two-way ANOVA	NpHR: 9; EYFP: 8	before/after	F = 0.395	dfn=1, dfd=30	EYFP, before vs. after	0.973	ns
						NpHR, before vs. after	0.947	ns
Supplementary Fig. 8d	Two way RM-ANOVA	NpHR: 9; EYFP: 8	S1, S2, S3	F =1.219	dfn=2, dfd=45	S1, EYFP vs. NpHR	0.833	ns
						S2, EYFP vs. NpHR	0.997	ns
						S3, EYFP vs. NpHR	0.987	ns
Supplementary Fig. 8e	Two-way ANOVA	EYFP: 8	Stimuli	F = 1.166		M1vs. O	0.463	ns

					dfn=2, dfd=42	M1vs. O	0.842	ns
						M2 vs. M1	0.682	ns
		NpHR: 9	Stimuli	F = 1.924	dfn=2, dfd=48	M1vs. O	0.392	ns
						M1vs. O	0.411	ns
						M2 vs. M1	0.405	ns
Supplementary Fig. 8f	Two-way ANOVA	NpHR: 9; EYFP: 8	EYFP/NpHR	F = 0.0169	dfn=1, dfd=30	EYFP, control vs. Sti	0.997	ns
						NpHR, control vs. Sti	0.938	ns
Supplementary Fig. 8g	Two way RM-ANOVA	NpHR: 9; EYFP: 8	S1, S2, S3	F = 3.659	dfn=2, dfd=45	S1, EYFP vs. NpHR	0.044	*
						S2, EYFP vs. NpHR	0.681	ns
						S3, EYFP vs. NpHR	0.0483	*
Supplementary Fig. 8h	Two-way ANOVA	EYFP: 8	Stimuli	F = 1.166	dfn=2, dfd=42	M1vs. O	0.394	ns
						M1vs. O	0.727	ns
						M2 vs. M1	0.593	ns
		NpHR: 9	Stimuli	F = 9.028	dfn=2, dfd=48	M1vs. O	0.011	*
						M1vs. O	0.047	*
						M2 vs. M1	0.025	*
Supplementary Fig. 9b	Two-way ANOVA	NpHR: 9; EYFP: 8	ON/OFF	F = 0.572	dfn=1, dfd=30	EYFP, ON vs. OFF	0.896	ns
						NpHR, ON vs. OFF	0.017	*
Supplementary Fig. 10a, %time	Two-way ANOVA with Bonferroni's Post-Hoc	EYFP: 8; NpHR: 7	S1	F = 8.844	dfn=1, dfd=26	EYFP, M1 vs. O	0.003	**
						NpHR, M1 vs. O	0.349	ns
			S2	F = 2.070	dfn=1, dfd=26	EYFP, M1 vs. O	0.147	ns
						NpHR, M1 vs. O	0.572	ns
			S3	F = 8.098	dfn=1, dfd=26	EYFP, M1 vs. M2	0.003	**
						NpHR, M1 vs. M2	0.419	ns
Supplementary Fig. 10a, transient rate	Two-way ANOVA with Bonferroni's Post-Hoc	EYFP: 8; NpHR: 7	S1	F = 3.699	dfn=1, dfd=26	EYFP vs. NpHR, M1	0.047	*
						EYFP vs. NpHR, O	0.667	ns
			S2	F = 16.81	dfn=1, dfd=26	EYFP vs. NpHR, M1	0.012	*
						EYFP vs. NpHR, O	0.997	ns
			S3	F = 1.935	dfn=1, dfd=26	EYFP vs. NpHR, M2	0.035	*
						EYFP vs. NpHR, M1	0.800	ns
		EYFP: 8	Stimuli			M1vs. O	0.025	*

Supplementary Fig. 10b	Two way-ANOVA			F = 0.474	dfn=2, dfd=42	M1vs. O	0.024	*
						M2 vs. M1	0.049	*
		NpHR: 7	Stimuli	F = 0.2009	dfn=2, dfd=36	M1vs. O	0.787	ns
						M1vs. O	0.810	ns
						M2 vs. M1	0.844	ns
Supplementary Fig. 10 c , %time	Two-way ANOVA with	EYFP: 8; NpHR: 7	S1	F = 3.971	dfn=1, dfd=26	EYFP, M1 vs. O	0.001	**
						NpHR, M1 vs. O	0.505	ns
			S2	F = 1.804	dfn=1, dfd=26	EYFP, M1 vs. O	0.375	ns
						NpHR, M1 vs. O	0.329	ns
			S3	F = 8.470	dfn=1, dfd=26	EYFP, M1 vs. M2	0.000	***
						NpHR, M1 vs. M2	0.441	ns
Supplementary Fig. 10 c, transient rate	Two-way ANOVA with	EYFP: 8; NpHR: 7	S1	F = 13.042	dfn=1, dfd=26	EYFP vs. NpHR, M1	0.021	*
						EYFP vs. NpHR, O	0.013	*
			S2	F = 9.830	dfn=1, dfd=26	EYFP vs. NpHR, M1	0.023	*
						EYFP vs. NpHR, O	0.053	ns
			S3	F = 9.109	dfn=1, dfd=26	EYFP vs. NpHR, M2	0.044	*
						EYFP vs. NpHR, M1	0.041	*
Supplementary Fig. 10 d	Two way-ANOVA	EYFP: 6	Stimuli	F = 1.661	dfn=2, dfd=42	M1vs. O	0.013	*
						M1vs. O	0.041	*
						M2 vs. M1	0.001	**
		NpHR: 6	Stimuli	F = 0.206	dfn=2, dfd=36	M1vs. O	0.543	ns
						M1vs. O	0.539	ns
						M2 vs. M1	0.969	ns
