# Supplementary material for: Pattern decorrelation in the mouse medial prefrontal cortex enables social preference and requires MeCP2
Source: Nat Commun. 2022 Jul 6;13:3899. doi: 10.1038/s41467-022-31578-9 (PMC9259602; doi:10.1038/s41467-022-31578-9)
Supplement: Supplementary file 3 — Description of Additional Supplementary Files [file 41467_2022_31578_MOESM3_ESM.pdf]

Title: Supplementary Movie 1.
Description: Female WT mouse prefers interacting with other mouse (M1) over object (O) during session 1.

Title: Supplementary Movie 2:
Description: Female Mecp2+/- mice lack the usual murine preference for interacting with other mouse (M1) over object (O) during session 1.

Title: Supplementary Movie 3.
Description: Female WT mouse prefers interacting with the new mouse (M2) over the old mouse (M1) during session 3.

Title: Supplementary Movie 4:
Description: Female Mecp2+/- mice lack the usual preference for interacting with a new mouse (M2) over the old mouse (M1) during session 3.
