# Supplementary material for: Pattern decorrelation in the mouse medial prefrontal cortex enables social preference and requires MeCP2
Source: Nat Commun. 2022 Jul 6;13:3899. doi: 10.1038/s41467-022-31578-9 (PMC9259602; doi:10.1038/s41467-022-31578-9)
Supplement: Supplementary file 8 — Reporting Summary [file 41467_2022_31578_MOESM8_ESM.pdf]

nature portfolio

Corresponding author(s): Hui Lu

Last updated by author(s): May 10, 2022

Reporting Summary

Nature Portfolio wishes to improve the reproducibility of the work that we publish. This form provides structure for consistency and transparency in reporting. For further information on Nature Portfolio policies, see our Editorial Policies and the Editorial Policy Checklist.

Statistics

For all statistical analyses, confirm that the following items are present in the figure legend, table legend, main text, or Methods section.

n/a	Confirmed	
☐	☒	The exact sample size (*n*) for each experimental group/condition, given as a discrete number and unit of measurement
☐	☒	A statement on whether measurements were taken from distinct samples or whether the same sample was measured repeatedly
☐	☒	The statistical test(s) used AND whether they are one- or two-sided<br>*Only common tests should be described solely by name; describe more complex techniques in the Methods section.*
☐	☒	A description of all covariates tested
☐	☒	A description of any assumptions or corrections, such as tests of normality and adjustment for multiple comparisons
☐	☒	A full description of the statistical parameters including central tendency (e.g. means) or other basic estimates (e.g. regression coefficient) AND variation (e.g. standard deviation) or associated estimates of uncertainty (e.g. confidence intervals)
☐	☒	For null hypothesis testing, the test statistic (e.g. *F*, *t*, *r*) with confidence intervals, effect sizes, degrees of freedom and *P* value noted<br>*Give P values as exact values whenever suitable.*
☒	☐	For Bayesian analysis, information on the choice of priors and Markov chain Monte Carlo settings
☒	☐	For hierarchical and complex designs, identification of the appropriate level for tests and full reporting of outcomes
☐	☒	Estimates of effect sizes (e.g. Cohen's *d*, Pearson's *r*), indicating how they were calculated

Our web collection on statistics for biologists contains articles on many of the points above.

Software and code

Policy information about availability of computer code

Data collection	nVista HD 2.1 (Inscopix) for calcium imaging acquisition<br>nVoke 2.0 (Inscopix) for optogenetic manipulation and calcium imaging simultaneously<br>Topscan-Topview behavior analyzing system (Clever Sys) for OFT, EPM, and social interaction tests<br>Confocal microscope (Zeiss LSM 710) for histological examination
Data analysis	Data processing software 1.2.1 (Inscopix) for neuron signal extraction<br>Python (v3.8) got calcium imaging data analysis, analysis of neural activity-behavior correlation, and statistical analysis<br>Topscan-Topview behavior analyzing system (Clever Sys) for behavioral data analysis<br>Prism 7 (GraphPad Software) and Excel (Microsoft) for statistical analysis and data plotting<br>SPSS statistics 24 (IBM) for statistical analysis

For manuscripts utilizing custom algorithms or software that are central to the research but not yet described in published literature, software must be made available to editors and reviewers. We strongly encourage code deposition in a community repository (e.g. GitHub). See the Nature Portfolio guidelines for submitting code & software for further information.

Data

Policy information about availability of data

All manuscripts must include a data availability statement. This statement should provide the following information, where applicable:

- Accession codes, unique identifiers, or web links for publicly available datasets
- A description of any restrictions on data availability
- For clinical datasets or third party data, please ensure that the statement adheres to our policy

All data and codes in the main text or the supplementary materials are available upon reasonable request.

Field-specific reporting

Please select the one below that is the best fit for your research. If you are not sure, read the appropriate sections before making your selection.

☒ Life sciences ☐ Behavioural & social sciences ☐ Ecological, evolutionary & environmental sciences

For a reference copy of the document with all sections, see nature.com/documents/nr-reporting-summary-flat.pdf

Life sciences study design

All studies must disclose on these points even when the disclosure is negative.

Sample size	The sample sizes for behavioral experiments were determined by the current standard used for mice in behavioral neuroscience experiments, based on the minimal number of mice required to detect significance with a α rate set to 0.05 in a standard-powered experiment with a statistical power of 80% or better.<br>The sample size for the number of neurons was 62~129 neurons expressing GCaMP6m for each individual mouse (mean =116), and was affected by the volume of viral GCaMP6m injected, level of expression and the efficiency of Cre-mediated recombination. After the processing of the imaging data (after neuron identification by principal component and independent component analyses (PCA-ICA)), 1~9 % of the identified components were discarded as artefacts and the remainings were treated as neurons and used for further analysis.
Data exclusions	All exclusion criteria were established prior to data collection:<br>(1) for combined imaging and behavioral experiments, mice were posthoc excluded if the lens was placed outside of the prelimbic cortex, or the imaging plane was unclear or occluded by blood or debris. (2) For combined imaging and optogenetic manipulation experiments, mice were excluded if they did not exhibit NpHR or EYFP expression in prelimbic regions, or major virus expression was detected outside of the prelimbic region, or the lens was not correctly placed. (3) For across-session analysis, the mice were excluded if the behavior of a stimulus interaction was lacked in one of three sessions.
Replication	All experiments reported here were reliably reproduced in independent experimental groups for behaviors, or several animals for calcium imaging. Multiple rounds of experimentation were required, i.e., from multiple mice, which were averaged for the presented datasets.
Randomization	In combined imaging and behavioral experiment, CamKII-Cre mice or PV-Cre mice were assigned to experimental groups based on their genotypes. In optogenetic experiments, PV-Cre Mecp2+/- (RTT) and Mecp2+/+ (WT) mice were randomly assigned to the EYFP and NpHR groups and two kinds of optogenetic manipulation-involved social tests were interleaved between two groups. In social behavioral test, social stimulus was randomized placed in one of the two lateral chamber in S1.
Blinding	Experimenters were blind to mouse genotype during experimental sessions. During data collection, the investigators were blinded to the groups of different genotypes or viruses, but not the groups of two manipulation methods for that the experimenter need to give different manipulations to whole-arena and location-specific groups. Analysis of behavioral and neural data was performed by an experimenter blinded to the group assignment of the animal. Computer-based analysis was used in the location-specific optogenetic manipulation and all the behavioral and neural data analyses.

Reporting for specific materials, systems and methods

We require information from authors about some types of materials, experimental systems and methods used in many studies. Here, indicate whether each material, system or method listed is relevant to your study. If you are not sure if a list item applies to your research, read the appropriate section before selecting a response.

Materials & experimental systems

n/a	Involved in the study
☒	☐ Antibodies
☒	☐ Eukaryotic cell lines
☒	☐ Palaeontology and archaeology
☐	☒ Animals and other organisms
☒	☐ Human research participants
☒	☐ Clinical data
☒	☐ Dual use research of concern

Methods

n/a	Involved in the study
☒	☐ ChIP-seq
☒	☐ Flow cytometry
☒	☐ MRI-based neuroimaging

Animals and other organisms

Policy information about studies involving animals; ARRIVE guidelines recommended for reporting animal research

Laboratory animals	All mice used in this study are obtained by breeding male mice with C57BL/6 background and female mice with 129S6SvEvTac background. Surgery with GRIN lens implantation were performed at 3.5 to 4 month of age. Behavioral test and calcium imaging were performed one month after the surgery. The genotype of Mecp2 mutant mice and wild-type littermates were confirmed using PCR-based genotyping in each litter.
Wild animals	The study did not involve wild animals.
Field-collected samples	The study did not involve samples that were collected in the field.
Ethics oversight	All procedures described in this paper were approved by the Institutional Animal Care and Use Committee of the George Washington University, and were in accordance with NIH Guidelines.

Note that full information on the approval of the study protocol must also be provided in the manuscript.
